# TWEAK Enhances E-selectin and ICAM-1 Expression, and May Contribute to the Development of Cutaneous Vasculitis

**DOI:** 10.1371/journal.pone.0056830

**Published:** 2013-02-15

**Authors:** Tao Chen, Zai-pei Guo, Li Li, Meng-meng Li, Ting-ting Wang, Rui-zhen Jia, Na Cao, Jing-yi Li

**Affiliations:** 1 Department of Dermatovenereology, West China Hospital of Sichuan University, Chengdu, Sichuan, China; 2 Open Laboratory, West China Second Hospital of Sichuan University, Chengdu, Sichuan, China; University of Illinois at Chicago, United States of America

## Abstract

Our previous work indicated that TWEAK is associated with various types of cutaneous vasculitis (CV). Herein, we investigate the effects of TWEAK on vascular injury and adhesion molecule expression in CV mice. We showed that TWEAK priming in mice induced a local CV. Furthermore, TWEAK priming also increased the extravasation of FITC-BSA, myeloperoxidase activity and the expression of E-selectin and ICAM-1. Conversely, TWEAK blockade ameliorated the LPS-induced vascular damage, leukocyte infiltrates and adhesion molecules expression in LPS-induced CV. In addition, TWEAK treatment of HDMECs up-regulated E-selectin and ICAM-1 expression at both mRNA and protein levels. TWEAK also enhanced the adhesion of PMNs to HDMECs. Finally, western blot data revealed that TWEAK can induce phosphorylation of p38, JNK and ERK in HDMECs. These data suggest that TWEAK acted as an inducer of E-selectin and ICAM-1 expression in CV mice and HDMECs, may contribute to the development of CV.

## Introduction

Vasculitis is characterized by an inflammatory reaction directed at vessels, in which destruction of the wall of blood vessels by leukocytes is the primary event. Aetiologically, cutaneous vasculitis (CV) can be classified as a primary phenomenon (such as Henoch-Schönlein purpura, urticarial vasculitis, allergic vasculitis, Behcet disease or nodular erythema), or as a secondary disorder [a manifestation of infection, adverse drug eruption, malignancies or connective tissue diseases, such as systemic lupus erythematosus (SLE), Sjögren's syndrome or rheumatoid arthritis]. Inflammatory injury to blood vessel walls plays a crucial role in the development of CV. Nevertheless, the molecular mechanisms underlying the perivascular leukocyte infiltrates and endothelial damage in these diseases are not yet fully understood.

Tumor necrosis factor-like weak inducer of apoptosis (TWEAK), which binds to its receptor fibroblast growth factor-inducible 14 (Fn14), is a member of the tumor necrosis factor (TNF) ligand superfamily. Recent evidence indicates that TWEAK is a multifunctional cytokine that regulates cell growth, angiogenesis, apoptosis and inflammation via activation of Fn14 [1]–[4]. Fn14 is highly expressed in endothelial cells. Moreover, it has been reported that TWEAK/Fn14 interaction plays a crucial role in many types of pathologic inflammatory disorders, such as atherosclerosis [5], diabetes [6], chronic kidney disease [7], cardiac dysfunction and failure [8] and SLE [9]. In these conditions, endothelial cells are the initial sites of inflammatory damage.

To investigate whether TWEAK/Fn14 interaction also play some roles in the pathogenesis of CV, some preliminary studies have been performed. Our previous publication [10] together with unpublished work indicated that TWEAK serum levels in patients with Henoch-Schönlein purpura, urticarial vasculitis and allergic vasculitis were markedly elevated. Furthermore, Fn14 is more abundantly expressed in the perivascular dermis of lesional skin in patients with these types of CV as compared with healthy controls. In line with this, a recent report showed that serum TWEAK levels were higher in SLE patients with vasculitis than those without vasculitis [11]. As a multifunctional cytokine, TWEAK mRNA is expressed in various tissues and cell lines [12]. In vitro, it has been reported that TWEAK can be released by inflammatory leukocytes, activated endothelial cells and activated platelets [13]–[15]. Therefore, it is plausible to propose that in CV, elevated serum concentration of TWEAK may result from excessive production by these cell types, which may interact with Fn14 on endothelial cells and play some roles in the pathogenesis of these diseases. Additionally, several recent studies have indicated that TWEAK may be an important mediator in tissue injury or inflammatory reaction [16]–[19]. In order to further extend our understanding about the roles of TWEAK/Fn14 interaction in the pathogenesis of CV and to develope a possible novel therapeutic strategy to interfere with vascular inflammation in CV, the direct roles of TWEAK application in skin and cultured human dermal microvascular endothelial cells (HDMECs) were investigated. Moreover, we also investigate the effects of TWEAK blockade in a Shwartzman reaction animal model system of CV (an important animal model being used extensively to test therapeutic approaches to vascular inflammation and injury) [20]–[22].

Adhesion molecules are complex membrane proteins located on the cell surface involved with intercellular binding and communication. Among them, E-selectin and intercellular adhesion molecule-1 (ICAM-1) are predominantly expressed on the surface of endothelial cells and play an essential role in local leukocyte recruitment to the vessel wall [23], [24]. In this study, we also investigated the effects of TWEAK on E-selectin and ICAM-1 expression in mice and cultured HDMECs.

## Materials and Methods

### Ethics statement

Ethical approval for the work was given by the University Committee on Use and Care of Laboratory Animals at Sichuan University.

### Animal model

Male BALB/c mice (8–10 weeks old, each of average weight 23 g) were obtained from Sichuan University animal centre (Sichuan, China). Local Shwartzman reaction was induced in BALB/c mice through s.c. injection of 7.5 µg of lipopolysaccharide (LPS, Sigma-Aldrich, St. Louis, MO, USA) into the left ear and after 24 h challenged with 150 µg LPS i.p., as previously reported [22], [25]. Control mice were treated with equal volume of phosphate-buffered saline (PBS) s.c. and i.p. at the same time.

For TWEAK priming, mice were injected s.c. with either 1 µg recombinant TWEAK (Peprotech, London, UK) or equal volume of PBS into the left ear and challenged with LPS 150 µg i.p. 24 h later.

For TWEAK blockade, mice were injected i.p. with either 200 µg neutralizing rat anti-mouse TWEAK monoclonal antibody (mAb) (MTW-1, Biolegend, San Diego, CA, USA)) or 200 µg matching isotype control (IC) mAb (IgG1, Biolegend, San Diego, CA, USA) 1 h after priming with LPS s.c. and then challenged with LPS 150 µg i.p.

### Measurement of serum levels of TWEAK

The serum levels of TWEAK from LPS-treated mice and control group were analyzed by ELISA using commercially available kits (R&D Systems Inc., Minneapolis, MN, USA), according to the manufacturer's instructions.

### Immunohistochemistry

Tissue samples from the mouse ears were formalin fixed, embedded in paraffin and cut onto glass slides. Immunohistochemical staining was performed as described [2], [22] using antibodies against TWEAK, Fn14, E-selectin or ICAM-1 (Beijing Biosynthesis, Beijing, China) followed by incubation with horseradish peroxidase-conjugated secondary antibodies.

### Evaluation for vascular damage

After treatment, the hemorrhagic lesions were analyzed by a semiquantitative score from 1 to 4 for the vascular hemorrhage (size and number of small hemorrhages) as described [25].

To quantify vessel damage, the amount of extravasated FITC-labeled bovine serum albumin (BSA) in the ears was also detected as described [25]. Mice were injected i.p. with FITC-BSA 2.5 mg 24 h after priming. 6 h after LPS challenge, tissue samples from the left ears and untreated right ears were collected and subjected to trypsin for 40 min. FITC fluorescence in the supernatant was quantitated spectrophotometrically. We compared the fluorescence intensity ratio from the left ear to the right ear in each sample from different groups.

### Analysis of myeloperoxidase (MPO) activity

MPO activity was assessed by measuring the H2O2 dependent oxidation of 3,3',5,5'-tetramethylbenzidine as described previously [26]. 6 h after systemic challenge with LPS, tissue samples were collected and homogenized. MPO activity was determined spectrophotometrically using commercially available kits (Nanjing Institute of Jiancheng Biological Engineering, Nanjing, China) according to the manufacturer's instructions.

### Cell culture

HDMECs were isolated from the foreskins as described [27] and grown on 1% gelatin-coated dishes in 5% CO2 humidified air at 37°C in endothelial cell basal medium supplemented with 10% heat-inactivated fetal bovine serum, 100 units/ml penicillin, 100 units/ml streptomycin, 35 µg/ml endothelial cell growth supplement, and 5 units/ml heparin. HDMECs cell line (HMEC-1) was obtained from the Centers for Disease Control and Prevention (CDC, Atlanta, GA, USA). Cells were maintained in RPMI-1640 supplemented with 10% heat-inactivated FBS, 100 units/ml penicillin, 100 units/ml streptomycin and 2 mM L-glutamine. HDMECs (passage 4–7) and HMEC-1cells (passage 10–15) were used for all experiments.

### Measurement of Fn14 expression in HDMECs

For surface expression of Fn14, cultured HDMECs were harvested with trypsin and washed with PBS. Then, 1×10^6^ cells were incubated with anti-Fn14 mAb (ITEM-4, Biolegend, San Diego, CA, USA)) or IC mAb for 1 h followed by a PE-labeled secondary antibody. Cells were then suspended in PBS and analyzed by flow cytometry for fluorescence.

### Analysis of cell apoptosis

Double staining for Annexin V FITC and propidium iodide was performed to evaluate the apoptotic rates of HMEC-1 cells. Cells were left un-treated or stimulated with TWEAK 500 ng/ml for 12 h. Then, cells were harvested with trypsin. 1×10^6^ cells were suspended in binding buffer and incubated with Annexin V FITC and propidium iodide for 25 minutes in the dark. After that, the fluorescence of each sample was determined by flow cytometry.

### Observation of intracellular ROS formation

Intracellular ROS formation in HDMECs was observed using cell-permeable, redox-sensitive dye DCFH-DA (Sigma-Aldrich, St. Louis, MO, USA). Cells were left un-treated or treated with TWEAK at concentrations ranging from 10 ng/ml to 500 ng/ml for various intervals. After treatment, cells were incubated with 10 µmol/L DCFH-DA for 0.5 h in the dark. After three washes, samples were observed and photographed by using fluorescence microscopy.

### Measurement of E-selectin and ICAM-1 protein expression in HDMECs

The protein expression of both E-selectin and ICAM-1 in HDMECs was determined by flow cytometry. Briefly, cells were treated with TWEAK at various concentrations, TNF-α 10 ng/ml, TWEAK 100 ng/ml plus anti-Fn14 mAb or TWEAK 100 ng/ml plus IC mAb for 6 h. Then, cells were harvested with trypsin. 1×10^6^ cells were stained with FITC labeled antibodies against E-selectin and ICAM-1 (Beijing Biosynthesis, Beijing, China) for 0.5 h. After washes, cells were suspended in PBS and analyzed by flow cytometry for fluorescence. Unless otherwise stated, data are shown as the mean fluorescence intensity of the sample minus that of a sample stained with an FITC labeled IC mAb.

To investigate whether TWEAK upregulated E-selectin and ICAM-1 protein expression through mitogen activated protein kinases (MAPKs) pathway, a separate study was carried out to evaluate the effects of various MAPKs inhibitors (SB203580, SP600125 and PD98059) on these adhesion molecules protein expression in TWEAK-induced HDMECs. Briefly, cells were pre-incubated with these MAPKs inhibitors and then exposed to TWEAK for 6 h. After that, cells were harvested and flow cytometry was performed as described earlier.

### Adhesion assay

The cell adhesion assay was performed as described [28]. The human polymorphonuclear leukocytes (PMNs) were isolated from heparinized peripheral blood of healthy human volunteers using commercially available kits (Tianjin Haoyang Biological Manufacture Co. Ltd., Tianjin, China) following the manufacturer's instructions. Then PMNs were labeled with BCECF-AM (Beyotime Institute of Biotechnology, Jiangsu, China) 10 µM for 0.5 h, washed, and resuspended in serum free media. HDMECs were then cultured in 24-well plates and treated as described above. After treatment, HDMECs were co-incubated with 1×0^5^ BCECF-AM labeled PMNs for 0.5 h. Nonadhering PMNs were removed and wells were washed with PBS. Samples were observed and photographed by using fluorescence microscopy. PMNs bound to HDMECs were lysed and the intensity of fluorescence of BCECF-AM in the lysates was then measured spectrophotometrically.

### Western blot analysis

The expression of phosphorylated p38, c-Jun NH2 -terminal kinase (JNK), extracellular signal-regulated kinase (ERK) and total p38, JNK, ERK in TWEAK-induced HDMECs was determined by western blot. Briefly, cells were treated with TWEAK 50 ng/ml for various intervals. After that, cells were washed with cold PBS and lysed using lysis buffer. Then, 30 µg protein samples were separated using 12% SDS-PAGE gels with electrophoresis and transferred to polyvinylidene fluoride membranes. Following treatment with Tris-buffered saline-Tween-20 including 5% BSA, the membranes were probed with the primary antibody (Cell Signaling Technology, Beverly, MA, USA) overnight followed by appropriate horseradish peroxidase-conjugated secondary antibody for 1 h. Bound antibodies were developed by chemiluminescence, according to the enhanced chemiluminescence protocol. The results were normalized to the GAPDH expression.

### Real-time quantitative PCR

The mRNA levels of E-selectin and ICAM-1 in TWEAK-induced HDMECs were measured by real-time quantitative PCR. Confluent monolayers of HDMECs were pre-treated with various MAPKs inhibitors (SB203580, SP600125 and PD98059) for 0.5 h followed by incubation with TWEAK 100 ng/ml for a further 4 h. After treatment, total RNA was extracted from cells using Trizol reagent (Invitrogen Corp, Carlsbad, CA, USA). cDNA was synthesized from 1 µg of total RNA by reverse transcription using revertAid™ First-strand cDNA Synthesis Kit (Fermentas, Vilnius, Lithuania). Each cDNA sample was amplified for the interested gene and GAPDH in a 25 µl reaction volume containing 12.5 µl Maxima™ SYBR Green qPCR Master Mix (Fermentas, Vilnius, Lithuania). The following primers were used: human E-selectin forward primer, 5'-AGAGGTTCCTTCCTGCCAAG-3'; human E-selectin reverse primer, 5'-CAGAGCCATTGAGGGTCCAT-3'; human ICAM-1 forward primer, 5'-GACTCCAATGTGCCAGGCTT-3'; human ICAM-1 reverse primer, 5'-TAGGTGCCCTCAAGATCTCG-3'; human GAPDH forward primer, 5'- CGGAGTCAACGGATTTGGTC-3'; human GAPDH reverse primer, 5'- CGGTGCCATGGAATTTGCCA-3'. The PCR was started at 95°C for 10 min (initial denaturation), followed by a 40-cycle amplification (denaturation at 95°C for 15 s, annealing/extension at 60°C for 60 s). The mRNA levels of E-selectin and ICAM-1 were normalized with those of GAPDH mRNA.

### Statistical analysis

Each experiment was performed at least three times. All results are expressed as mean ± SD, Statistical differences between groups were determined according to one-way analysis of variance or Mann–Whitney Test. P<0.05 was considered statistically significant.

## Results

### Serum TWEAK levels and TWEAK receptor Fn14 expression are up-regulated in LPS-induced CV

**Figure 1:**
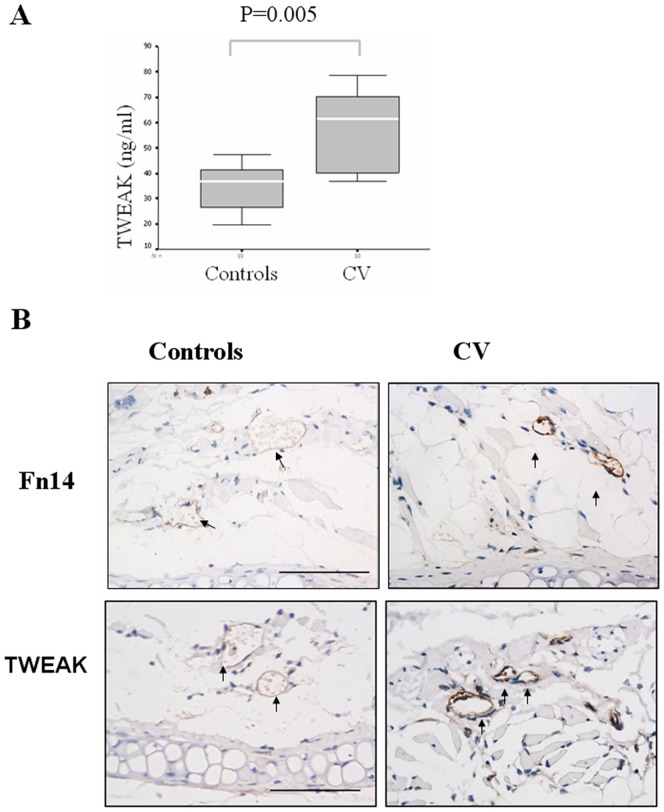
TWEAK and Fn14 are upregulated in LPS-induced CV. BALB/c mice were treated with LPS or equal volume of PBS s.c. into the left ear and challenge with LPS 150 µg or PBS i.p. respectively 24 h later. 6 h after challenge, sera and tissue samples were collected. (ten mice per group). (A) Serum TWEAK levels were determined by ELISA. Values are expressed as Mean ± SD. *P*-values are based on the Mann–Whitney *U*-test. (B) The expression of TWEAK and its receptor Fn14 on endothelial cells in the skin were detected by immunohistochemistry. Scale bars, 50 µm.

Firstly, we investigated serum TWEAK levels and the expression of TWEAK and its receptor Fn14 in mice when CV was elicited by LPS. Sera collected on 6 h after LPS challenge were assayed for TWEAK levels and compared with those in controls (PBS-treated mice) by ELISA. As presented in Figure 1A, TWEAK levels in LPS-treated mice were significantly higher than those in controls. By immunohistochemistry, we showed that TWEAK and Fn14 was expressed on endothelial cells in the skin from control mice. Furthermore, there was a substantial increase in TWEAK and Fn14 expression on the endothelium of blood vessels in LPS-induced CV mice (Figure 1B). Data suggests that TWEAK and Fn14 might be associated with LPS-induced CV.

### TWEAK injection induces a local CV

**Figure 2:**
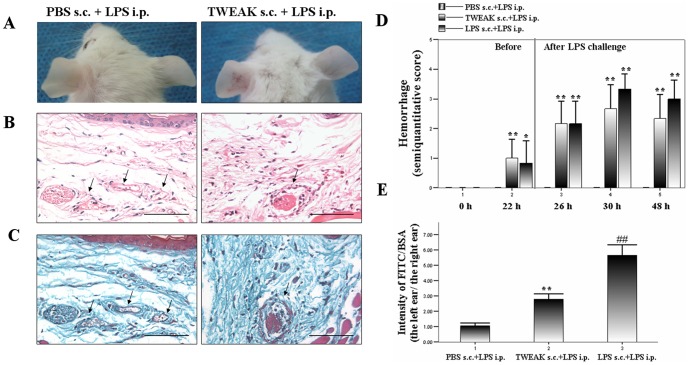
TWEAK priming induces a local CV. Mice were treated as described (six mice per group). (A) 6 h after LPS challenge, the hemorrhagic lesions in the ears were observed in TWEAK priming group. Tissue samples were stained for hematoxylin–eosin (B) and Masson's trichrome (C). Representative images were shown from three experiments. Scale bars, 50 µm. (D) The number and size of hemorrhagic lesions were analyzed according to the semiquantitative score as described^ 21^ at different time points. (E) Increased FITC-BSA extravasation was found in the left ear from TWEAK priming group and LPS priming group. Values are expressed as Mean ± SD; Mann–Whitney *U*-test *P<0.05, **P<0.01, compared with PBS priming group; ##P<0.01, compared with TWEAK priming group.

It has been reported that TWEAK i.p. injection into mice can enhance vascular and renal injury severity [29], we assessed the effects of local TWEAK injection by replacing the LPS priming. As shown in Figure 2A, when mice were primed with TWEAK and challenged with LPS 24 h later, purpuric rashes at the site of priming were moderately elicited. Moreover, we observed the typical necrotizing vasculitis characterized by neutrophils and a few lymphocytes infiltration, nuclear dust, and fibrinoid necrosis in TWEAK priming group similar to those found in LPS priming group (Figure 2B and 2C). In contrast, PBS priming did not result in hemorrhage or local CV. Moreover, we found that both TWEAK and LPS priming significantly increased the semiquantitative score for hemorrhage after priming for 22, 26, 30 and 48 h. (Figure 2D). To further confirm the effects of TWEAK priming on vascular damage, the extravasation of FITC-BSA was measured in the ears 6 h after FITC-BSA injection. As shown in Figure 2E, the extravasation of FITC-BSA was markedly elevated in TWEAK priming group. However, the extravasation of FITC-BSA in the ears in TWEAK priming group was significantly less than those in LPS priming group.

### TWEAK blockade reduces disease severity

**Figure 3:**
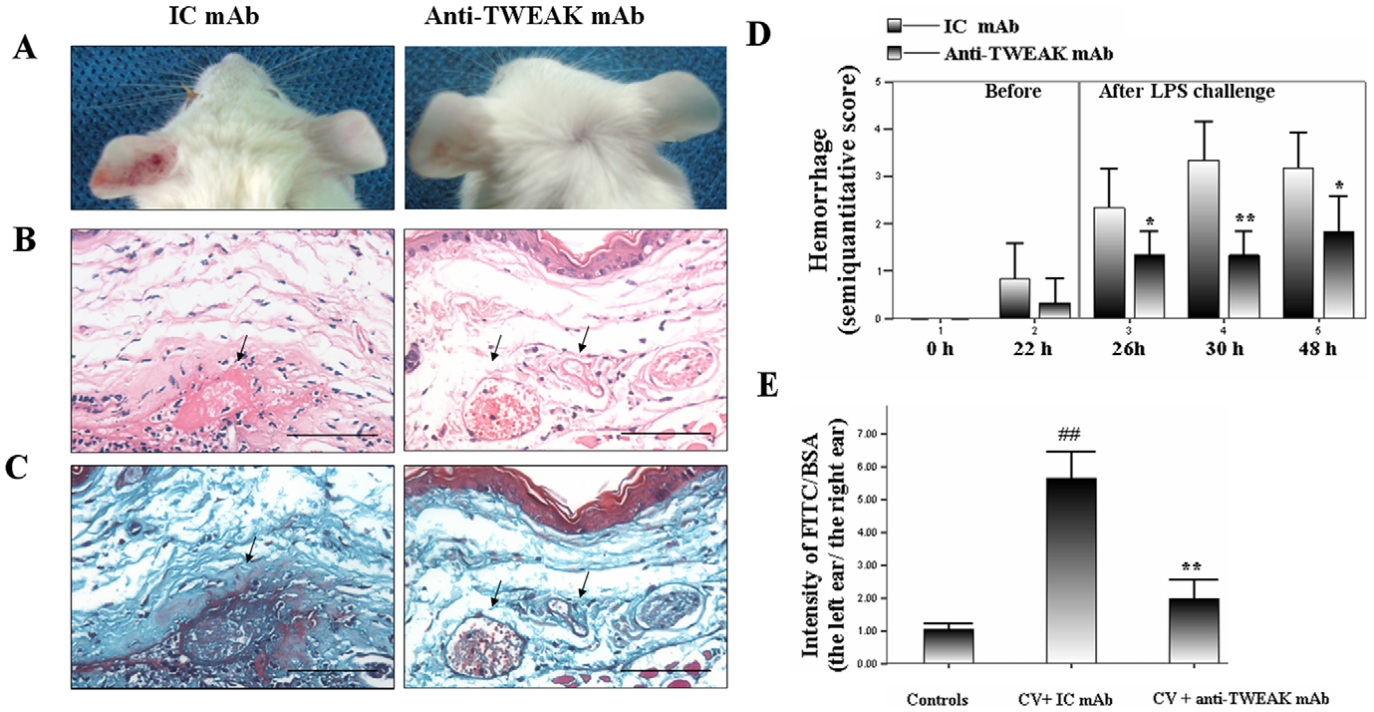
TWEAK neutralization blocks LPS induced CV. Mice were treated as described (six mice per group). (A) 6 h after systemic LPS challenge, the representative macroscopic appearance of the hemorrhagic lesions in the ears was shown. Tissue samples were collected and stained for hematoxylin–eosin (B) and Masson's trichrome (C). Scale bars, 50 µm. (D) The hemorrhagic lesions in different group were determined by a semiquantitative score as described^ 21^ at the indicated time points. (E) Extravasation of FITC-BSA was assessed spectrophotometrically in the ear from CV mice treated with anti-TWEAK mAb or IC mAb or PBS-treated mice (control group). Values are expressed as Mean ± SD; Mann–Whitney *U*-test *P<0.05, **P<0.01, compared with IC mAb-treated group; ##P<0.01, compared with control group.

In order to test whether blocking TWEAK activity is beneficial, animals were treated with either neutralizing anti-TWEAK mAb or IC mAb 1 h after priming. As presented in Figure 3A, 3B and 3C, 6 h after LPS challenge, anti-TWEAK mAb strikingly attenuated the clinical and histopathologic changes in LPS-induced CV mice when compared with IC mAb-treated group. Furthermore, we found that anti-TWEAK mAb markedly reduced the semiquantitative score for hemorrhage at 26, 30 and 48 h after LPS priming (Figure 3D). In addition, we also indicated that the extravasation of FITC-BSA was increased in CV mice treated with IC mAb relative to normal controls. However, anti-TWEAK mAb significantly suppressed LPS-induced extravasation of FITC-BSA (Figure 3E).

**Figure 6:**
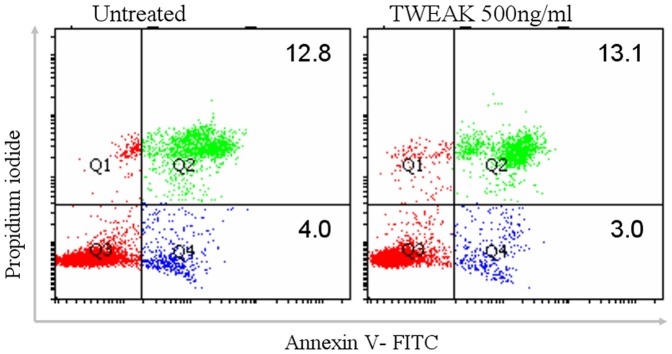
TWEAK does not induce cell apoptosis in HMEC-1 cells. Cells were treated with TWEAK 500 ng/ml for 12 h. After treatment, cells were suspended in binding buffer and double-stained with FITC-labled Annexin V and propidium iodide. Apoptotic rate of HDMECs were determined by flow cytometry.

### TWEAK priming enhances adhesion molecules expression and MPO activity

**Figure 4:**
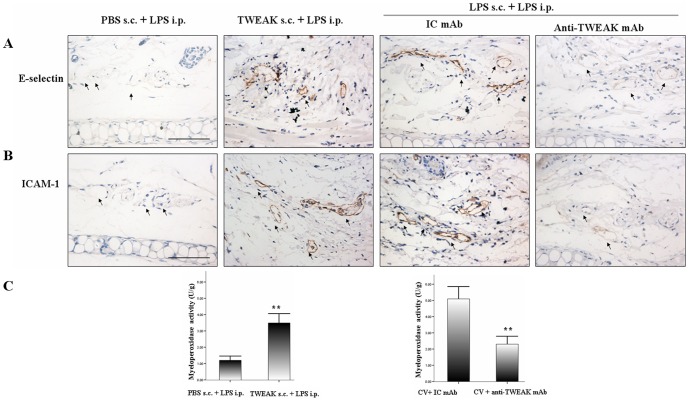
TWEAK priming upregulates but TWEAK blockade suppresses adhesion molecules expression and MPO activity. Mice were treated as described above, tissue sample were collected 6 h after systemic LPS challenge (six mice per group). Skin sections were stained with antibodies against E-selectin (A) and ICAM-1 (B). These observations were representative of multiple experiments and microscopic fields. Scale bars, 10 µm. (C) MPO activity was assessed for the quantification of PMNs accumulation in tissue samples by a colorimetric assay. Values are expressed as Mean ± SD; Mann–Whitney *U*-test **P<0.01.

As mentioned above, one of the important events in the development of vascular inflammation is the expression of the adhesion molecules on endothelial cells. In this study, we investigated the effects of TWEAK priming on E-selectin and ICAM-1 expression in mice. As shown in Figure 4A and 4B, TWEAK priming evidently up-regulated E-selectin and ICAM-1 expression on the endothelium of blood vessels relative to PBS priming group.

In addition, PMNs accumulation was also measured by quantifying deposition of MPO by infiltrated cells. As presented in Figure 4C, TWEAK priming also enhanced PMNs recruitment at the site of priming as observed by increased MPO activity.

### TWEAK blockade attenuates adhesion molecules expression and MPO activity in LPS-induced CV

In order to further elucidate the association between TWEAK and vascular inflammation, we investigated the ability of TWEAK blockade on adhesion molecules expression and MPO activity in LPS-induced CV. We found that anti-TWEAK mAb markedly blocked the vascular expression of E-selectin and ICAM-1 (Figure 4A and 4B) in LPS-induced CV. Moreover, we demonstrated that anti-TWEAK mAb also decreased MPO activity in the ears induced by LPS (Figure 4C).

### TWEAK receptor Fn14 is expressed in HDMECs

**Figure 5:**
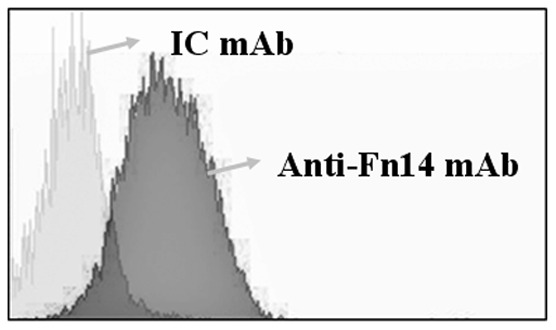
Fn14 is expressed in HDMECs. Cells were stained for Fn14 expression using mouse anti-Fn14 mAb (dark grey histogram) or IC mAb (light grey histogram) and PE-labeled secondary antibody. After that, Cells were resuspended in PBS and analyzed by flow cytometry for fluorescence.

To better establish the relevance of TWEAK/Fn14 pathway in CV, we investigated the direct effect of TWEAK on HDMECs in vitro. A previous report demonstrated that TWEAK receptor Fn14 is highly expressed in endothelial cells, including HDMECs [30]. By flow cytometry analysis, we also established the expression of Fn14 in HDMECs (Figure 5).

### TWEAK does not induce apoptosis and intracellular ROS production in HMEC-1 cells

It has been reported that TWEAK can stimulate apoptosis in certain cell types [31]. Moreover, the apoptotic cell death has been found in vascular endothelial cells in Shwartzman reaction [32], [33]. We asked ourselves whether the hemorrhagic activity induced by TWEAK in mice is due to cytotoxicity of TWEAK on HDMECs. However, there were no changes in the apoptosis and necrosis rates in HMEC-1 cells following stimulation with TWEAK 500 ng/ml for 12 h through Annexin V FITC and propidium iodide staining (Figure 6).

It is well established that oxidative stress is involved in the development of many types of CV [34], [35]. Moreover, some studies indicated that activation of endothelial cells by several members of the TNF ligand superfamily, such as TNF-α or CD40L, can induce intracellular ROS production [36], [37]. We next tested whether TWEAK-induced vascular damage could be attributed to ROS overproduction in endothelial cells. Whereas, we found that TWEAK (10–500 ng/ml) did not induce ROS formation by immunofluorescence staining of DCFH-DA (data not shown).

### TWEAK up-regulates E-selectin and ICAM-1 expression in HDMECs

**Figure 7:**
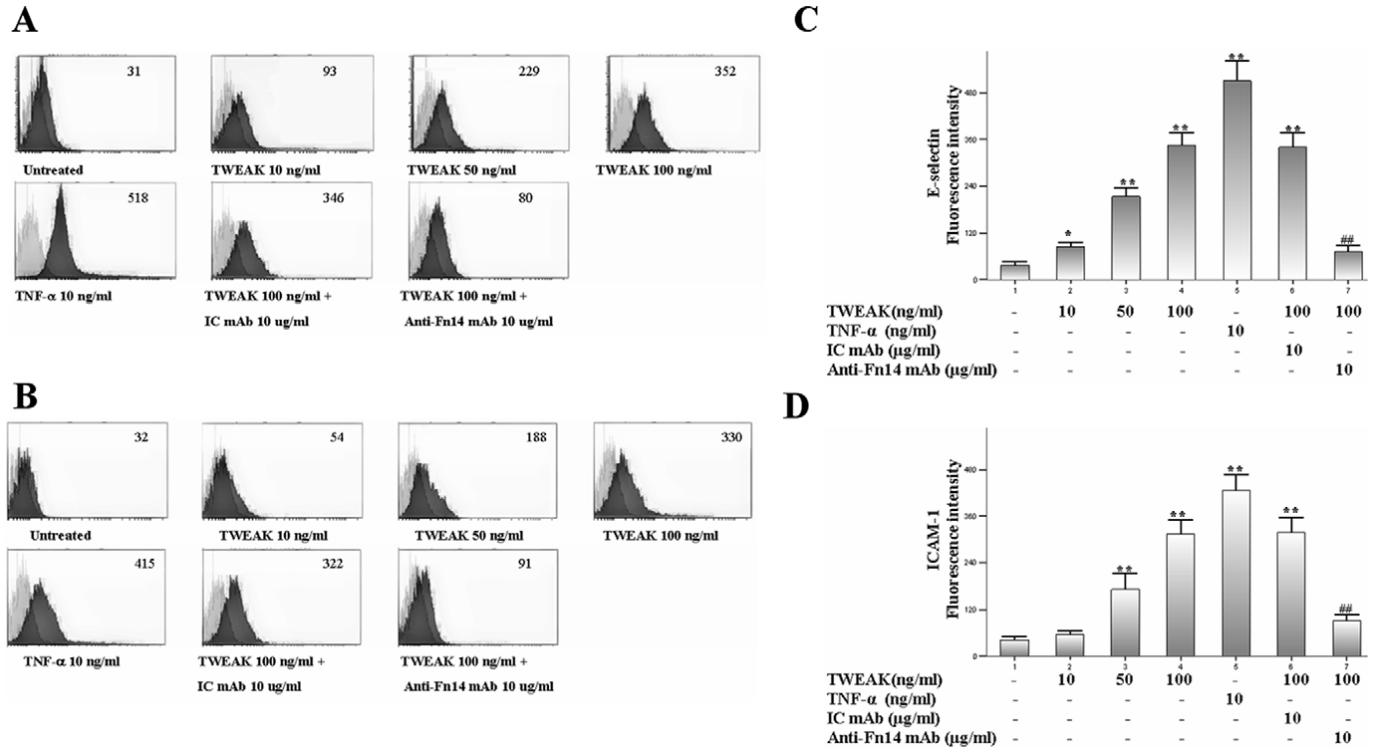
TWEAK enhances adhesion molecule expression in HDMECs. Cells were treated with TWEAK (1–100 ng/ml), TNF-a 10 ng/ml, TWEAK 100 ng/ml plus anti-human Fn14 mAb or IC mAb for 6 h. After treatment, cells were stained with FITC-labeled antibodies against E-selectin and ICAM-1. The expression of E-selectin (A and C) and ICAM-1 (B and D) were quantitatively analyzed by flow cytometry and depicted in the dark grey histograms. Cells stained with FITC-labeled IC mAb were depicted in the light grey histograms. Values are expressed as Mean ± SD; one-way analysis of variance n = 4.*P<0.05, **P<0.01, compared with untreated group; ##P<0.01, compared with TWEAK 100 ng/ml-treated group.

As local TWEAK injection can enhance E-selectin and ICAM-1 expression on the endothelium of blood vessels in mice, we then investigate the effects of TWEAK on these adhesion molecules expression in vitro. By flow cytometry analysis, we showed that TNF-α strongly and TWEAK moderately up-regulated both E-selectin (Figure 7A and 7C) and ICAM-1 (Figure 7B and 7D) protein expression. However, anti-Fn14 mAb markedly blocked the TWEAK-induced E-selectin and ICAM-1 expression.

### TWEAK enhances the adhesion of leukocytes to HDMECs

**Figure 8:**
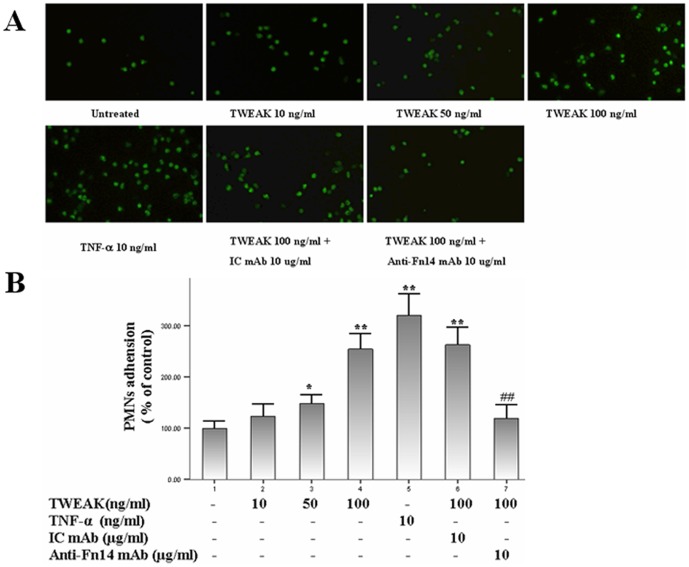
TWEAK induces the adhesion of human PMNs to HDMECs. Cells were treated with TWEAK (1–100 ng/ml), TNF-a 10 ng/ml or TWEAK 100 ng/ml plus anti-human Fn14 mAb or IC mAb for 6 h, and then co-incubated with fluorescent-labeled PMNs for 0.5 h. After treatment, PMNs bound to HDMECs were lysed. Fluorescent intensities of cell lysate were counted on a spectrofluorometer. Values are expressed as Mean ± SD; one-way analysis of variance n = 4. *P<0.05, **P<0.01, compared with untreated group; ##P<0.01, compared with TWEAK 100 ng/ml-treated group.

Because TWEAK significantly induced the adhesion molecules expression, we investigate whether the adhesion of leukocytes to HDMECs could also be enhanced by TWEAK-stimulated HDMECs. As presented in Figure 8, the adhesion of the human PMNs to HDMECs was significantly increased after TWEAK treatment for 6 h. However, in the presence of anti-Fn14 mAb the adhesion of PMNs to TWEAK-stimulated HDMECs was almost completely blocked.

### TWEAK induces the activation of MAPKs family proteins

**Figure 9:**
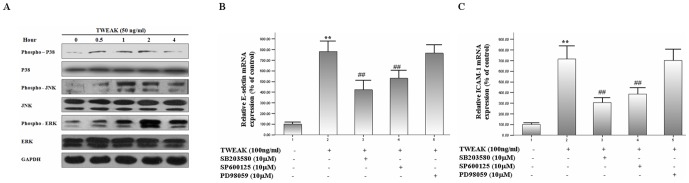
TWEAK activates MAPKs family proteins in HDMECs. (A) Cells were treated with TWEAK 50 ng/ml for various intervals. After treatment, cells were lysed, and 30 µg of protein samples were used for western blots analysis of the indicated proteins. Representative blots were shown from three experiments. (B and C) Cells were pre-treated with various MAPKs inhibitors (SB203580, SP600125 and PD98059) for 0.5 h followed by incubation with TWEAK 100 ng/ml for a further 4 h. The mRNA expression levels of E-selectin (B) and ICAM-1 (C) were determined by real-time quantitative PCR. Values are expressed as Mean ± SD; one-way analysis of variance n = 4. **P<0.01, compared with untreated group; ##P<0.01, compared with TWEAK -treated group.

A number of investigators have also reported that the activation of MAPKs, such as p38, JNK and ERK, contribute to the expression of adhesion molecules [38]. To explore the mechanisms by which TWEAK activated HDMECs, western blots were performed to evaluate the phosphorylated p38, JNK, ERK and total p38, JNK, ERK protein expression levels in TWEAK-induced HDMECs. As presented in Figure 9A, the increased phosphorylation of p38, JNK and ERK protein levels were observed in HDMECs treated with TWEAK 50 ng/ml at different time point.

**Figure 10:**
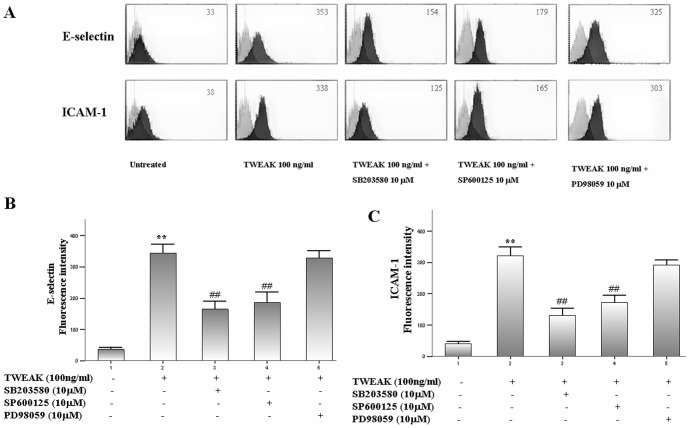
SB203580 and SP600125 but not PD98059 block TWEAK-induced adhesion molecules protein expression. Cells were pre-treated with various MAPKs inhibitors (SB203580, SP600125 and PD98059) for 0.5 h followed by incubation with TWEAK 100 ng/ml for a further 6 h. After treatment, cells were stained with FITC-labeled antibodies against E-selectin and ICAM-1. The expression of E-selectin (A and B) and ICAM-1 (A and C) were quantitatively analyzed by flow cytometry and depicted in the dark grey histograms. Cells stained with FITC-labeled IC mAb were depicted in the light grey histograms. Values are expressed as Mean ± SD; one-way analysis of variance n = 4. **P<0.01, compared with untreated group; ##P<0.01, compared with TWEAK 100 ng/ml-treated group.

To further dissect the involvement of MAPKs pathways in TWEAK-induced adhesion molecules expression in HDMECs, real-time quantitative PCR was used to analyze the effects of p38, JNK and ERK inhibitor on E-selectin and ICAM-1 expression in TWEAK-induced HDMECs. As shown in Figure 9B and 9C, exposure of cells to TWEAK 100 ng/ml for 4 h caused a significant increase in the mRNA levels of these adhesion molecules in HDMECs. However, pretreatment with SB203580 (a p38 inhibitor) and SP600125 (a JNK inhibitor), but not PD98059 (an ERK inhibitor) attenuated these changes. In addition, by flow cytometry analysis, we also indicated that SB203580 and SP600125 but not PD98059 can inhibit TWEAK-induced adhesion molecules expression at protein levels in HDMECs (Figure 10). These results suggest that TWEAK may act as a regulator of inflammatory response in HDMECs, and contribute to the adhesion molecules expression through p38 and JNK pathways.

## Discussion

CV comprises a wide spectrum of diseases that affect the blood vessels of the skin. Despite the different causes such as vascular deposition of immune complexes or activation by infection, the common pathophysiological feature is endothelial damage and perivascular leukocyte infiltrates. In this study, we indicated that TWEAK injection in skin followed by systemic LPS challenge induced a local CV. Moreover, TWEAK injection also enhanced the extravasation of FITC-BSA and MPO activity in mice. Conversely, by utilizing anti-TWEAK mAb, we found that TWEAK blockade effectively ameliorated the LPS-induced vascular damage and leukocyte infiltrates. In addition, in our unpublished work, similar effects were obtained by using anti-Fn14 mAb. These results suggest that TWEAK may be crucial for the development of vascular inflammation in skin.

Then we investigated the potential molecular mechanisms underlying TWEAK-induced vascular injury and inflammation.

Firstly, we demonstrated that TWEAK did not induce cell apoptosis and intracellular ROS production in HMEC-1 cells. In addition, in a supplementary study, we found that TWEAK injection in the ear did not lead to apoptotic endothelial cell death (data not shown). Actually, it has been recognized that TWEAK is a potent inducer of endothelial cell growth and survival and has anti-apoptosis effects in endothelial cells [30], [39], [40]. Hence we suggest that the TWEAK-induced vascular damage is unlikely due to the direct cytotoxic effects of TWEAK on HDMECs.

Ample evidence showed that the up-regulated adhesion molecules are a central event in triggering of vascular inflammation and the crucial contributors in the development of CV [22], [25]. In this study, we showed that TWEAK priming upregulated vascular E-selectin and ICAM-1 expression in the skin. Otherwise, TWEAK blockade markedly reduced E-selectin and ICAM-1 expression on the endothelium of blood vessels in LPS-induced CV. Furthermore, our in vitro experiments indicated that TWEAK up-regulated the expression of E-selectin and ICAM-1 in cultured HDMECs. As adhesion molecules are responsible for the leukocyte-endothelial cell adhesion, we also investigated the activity of TWEAK-treated HDMECs on leukocyte adhesion. We found that adhesion of PMNs to HDMECs was markedly increased after TWEAK treatment. These data suggest that TWEAK may exert its pro-inflammatory effects on HDMECs at least partly by inducing adhesion molecules expression and subsequent adhesion of leukocytes to endothelial cells. Moreover, by utilizing anti-human Fn14 mAb that blocks the TWEAK/Fn14 interaction, we demonstrated that Fn14 was expressed on HDMECs and almost completely mediated the pro-inflammatory effects of TWEAK.

MAPKs are serine-threonine protein kinases that regulate a variety of cellular processes, including growth, metabolism, apoptosis, and inflammation. The pivotal roles of certain MAPKs pathways, which are activated by TWEAK treatment, in certain cell types have been documented. For example, Donohue et al. reported that TWEAK treatment of human umbilical vein endothelial cells increased the levels of phosphorylated ERK and JNK [39]. Li et al. showed that TWEAK can induce phosphorylation of ERK, p38 and JNK in C2C12 myotubes [41]. Similarly, in our study, TWEAK was shown to phosphorylate ERK, p38 and JNK in HDMECs. Moreover, using specific inhibitors, we demonstrated that SB203580 and SP600125 but not PD98059 significantly inhibited E-selectin and ICAM-1 mRNA expression in TWEAK-activated HDMECs. Therefore, one mechanism by which TWEAK promoted adhesion molecules production in HDMECs may be responsible for the activation of p38 and JNK pathways. In addition, in a supplementary study, we demonstrated that blocking both p38 and JNK together caused a further but not complete inhibition (about 75% and 80% inhibition, respectively) of E-selectin and ICAM-1 mRNA expression in TWEAK-induced HDMECs. Hence, another pathway might be necessary for, or involved in, TWEAK-induced adhesion molecules expression.

Our previous work [10] showed that TWEAK can induce NF-κB activation and chemokines (including CCL5 and CXCL8) production in HMEC-1 cells. Moreover, increasing evidence indicates that TWEAK injection in mice can enhance chemokine production and promote chemotaxis of infiltrating cells [29], [42]. Thus, we suggest, as well as regulating the adhesion molecules expression, TWEAK may contribute to the development of vascular inflammation by inducing chemokine production.

Taken together, our findings provide new insights into the pathogenesis of CV. We report firstly that the TNF family member TWEAK may be one of essential mediators for the development of vascular inflammation in skin. We show that the pro-inflammatory effects of TWEAK in vessels are associated with the induction of E-selectin and ICAM-1 expression in HDMECs. Furthermore, this study provides the first evidence that TWEAK blockade substantially reduced vascular damage and perivascular leukocyte infiltrates in LPS-induced CV. It suggests that TWEAK may be a novel therapeutic target for leukocyte-endothelial cells adhesion in CV. In addition, targeting TWEAK may be of clinical benefit in the treatment of CV with renal involvement or joint involvement, as its receptor is abundantly expressed in these sites [16], [18], [43].
